# An ELISA Platform for the Quantitative Analysis of SARS-CoV-2 RBD-neutralizing Antibodies As an Alternative to Monitoring of the Virus-Neutralizing Activity

**DOI:** 10.32607/actanaturae.11776

**Published:** 2022

**Authors:** N. N. Kostin, T. V. Bobik, G. A. Skryabin, M. A. Simonova, V. D. Knorre, V. A. Abrikosova, Y. A. Mokrushina, I. V. Smirnov, N. L. Aleshenko, N. A. Kruglova, D. V. Mazurov, A. E. Nikitin, A. G. Gabibov

**Affiliations:** Shemyakin-Ovchinnikov Institute of Bioorganic Chemistry of the Russian Academy of Sciences, Moscow, 117997 Russia; Central Clinical Hospital of the Russian Academy of Sciences, Moscow, 117593 Russia; Institute of Gene Biology Russian Academy of Sciences, Moscow, 119334 Russia

**Keywords:** Gam-COVID-Vac, Sputnik V, CoviVac, virus-neutralizing activity, antibodies, SARS-CoV-2, COVID-19

## Abstract

Monitoring of the level of the virus-neutralizing activity of serum immunoglobulins ensures that one can reliably assess the effectiveness of any protection against the SARS-CoV-2 infection. For SARS-CoV-2, the RBD-ACE2 neutralizing activity of sera is almost equivalent to the virus-neutralizing activity of their antibodies and can be used to assess the level of SARS-CoV-2 neutralizing antibodies. We are proposing an ELISA platform for performing a quantitative analysis of SARS-CoV-2 RBD-neutralizing antibodies, as an alternative to the monitoring of the virus-neutralizing activity using pseudovirus or “live” virus assays. The advantage of the developed platform is that it can be adapted to newly emerging virus variants in a very short time (1–2 weeks) and, thereby, provide quantitative data on the activity of SARS-CoV-2 RBD-neutralizing antibodies. The developed platform can be used to (1) study herd immunity to SARS-CoV-2, (2) monitor the effectiveness of the vaccination drive (revaccination) in a population, and (3) select potential donors of immune plasma. The protective properties of the humoral immune response in hospitalized patients and outpatients, as well as after prophylaxis with the two most popular SARS-CoV-2 vaccines in Russia, were studied in detail using this platform. The highest RBD-neutralizing activity was observed in the group of hospitalized patients. The protective effect in the group of individuals vaccinated with Gam-COVID-Vac vaccine was 25% higher than that in outpatients and almost four times higher than that in individuals vaccinated with the CoviVac vaccine.

## INTRODUCTION

As of July 2022, more than 564 million people have been infected by the SARS-CoV-2 coronavirus and more than 6.3 million people have died from the COVID-19 infection all over the world [1]. Since the start of the pandemic, several dozen vaccines approved by the WHO [2, 3] and therapeutic antibodies [4 , 5, 6] have been developed. The vaccines were engineered on the basis of various platforms: protein subunits, viral vectors, RNA, DNA, inactivated viruses, etc. Unfortunately, the evaluation of the efficacy of the developed vaccines was impeded by the differences in the platforms, antigens, as well as immunologic assays and parameters used to assess the immune response. In late 2020, the WHO, the National Institute for Biological Standards and Control (NIBSC), and the Coalition for Epidemic Preparedness Innovations (CEPI) elaborated and distributed the International Standard for human anti-SARS-CoV-2 immunoglobulin (the NIBSC code: 20/136) [7]. The standard is a freeze-dried pool of plasma from 11 donors with a previous history of COVID-19; the pool has a neutralizing antibody activity of 1,000 international units per milliliter (IU/ml) and contains 1,000 binding antibody units per milliliter (BAU/ml). The elaboration of this standard has reduced the interlaboratory variability and provided a common language for data presentation, which is important for developing diagnostics, vaccines, and therapeutic antibodies, as well as for donor selection [8]. The level of virus-neutralizing activity of serum immunoglobulins ensures reliable assessment of the level of protection one enjoys against the SARS-CoV-2 infection. Considerable time and financial resources are necessary in studies that use the live virus to obtain quality data. The efforts of many researchers have recently focused on the development of quantitative procedures that are alternative to the existing platforms, where samples of the live SARS-CoV-2 virus are used [9, 10, 11, 12, 13].

In this work, we have studied the humoral response in individuals who received the most popular prophylactic vaccines in the Russian Federation – Gam-COVID-Vac (rAd26/rAd5, brand name Sputnik V) [14] and CoviVac (the inactivated virus) [15] – compared it to the antibody response in patients who had had mild and severe COVID-19, and analyzed the correlation between RBD and virus neutralization. As a result, we have proposed a platform for the quantitative analysis of SARS-CoV-2 RBD-neutralizing antibodies, as an alternative to monitoring the virus-neutralizing activity.

## EXPERIMENTAL

**Quantitative determination of RBD-specific IgG and identification of their isotypes by ELISA**

To perform a quantitative determination of RBD-specific IgG, 100 µl of a PBS solution of recombinant RBD (amino acid residues 320–537) produced in CHO cells (1 μg/ml) were added to the wells of MaxiSorp 96-well plates (Nunc, Denmark) and the plate was incubated overnight at 2–8°C. The unoccupied binding sites were then blocked by adding 150 μl of blocking buffer (PBS, 0.05% Tween-20, 0.1% sodium caseinate) into each well and incubating the plate at room temperature for 1 h. Serum samples in the blocking buffer were prepared in three dilutions (1 : 10, 1 : 50, 1 : 250) and three replicates in a separate 96-well plate with low sorption capacity. WHO primary standard solutions (NIBSC code: 20/136) and solutions of the secondary standard (obtained in the laboratory from a pool of serum samples collected from individuals who had had COVID-19 and characterized with respect to the primary standard) were prepared in the same plate in a blocking buffer in seven sequential threefold serial dilutions. Next, the serum samples and standards (100 μl/well) were added to the wells containing adsorbed RBD and incubated for 30 min in a thermo-shaker at 37°C, 700 rpm. After the incubation, the plate was washed five times by adding 350 μl of PBST (PBS, 0.05% Tween-20) to each well and 100 μl of horseradish peroxidase-conjugated anti-human IgG antibodies (Biosan, Novosibirsk, Russia, Cat. # I-3021) diluted 1 : 10 000 in a blocking buffer were then added to each well. After 30-min incubation (37°C, 700 rpm) and washing, 100 μl of the substrate TMB solution was added to each well and the plate was incubated for 15 min in the dark. The enzymatic reaction was stopped by adding 10% of the solution of orthophosphoric acid, and optical density (OD) in the wells at a wavelength of 450 nm (OD_450_) was measured on a plate spectrophotometer. The curves showing the mean OD value as a function of the concentration of RBD-specific IgG in the standards (BAU/ml) were plotted using the GraphPad Prism 8 software (USA). These curves were used to calculate the concentrations of RBD-specific IgG in the serum samples: the dilution of the sample whose mean OD_450_ lay in the OD_450_ range of the curve of the standard solution was chosen, and the resulting value (in BAU/ml) was multiplied by the respective dilution. The subclasses of RBD-specific IgG were analyzed according to the protocol described above, even though the calibration curves were not plotted, and the horseradish peroxidase conjugates of the following antibodies were used: anti-human IgG1 antibodies (HyTest, Finland, Cat. # 1G2cc), anti-human IgG2 antibodies (HyTest, Finland, Cat. # 1G5), anti-human IgG3 antibodies (HyTest, Finland, Cat. # 1G3cc), and anti-human IgG4 antibodies (HyTest, Finland, Cat. # 1G4cc). ELISA of IgG against nucleocapsid and linear antigens was carried out according to the procedure reported in [16]. The detection limit in the quantitative and qualitative assays of RBD-specific IgG was determined as follows: the mean OD_450_ value in the negative samples plus three standard deviations from the mean value in the negative samples.

**Determining the neutralizing activity for the live virus**

The neutralizing activity of the blood serum samples was determined in a neutralization reaction (NR) in which the formation of negative colonies produced by the SARS-CoV-2 virus in a 24-h-old monolayer of Vero C1008 cells under agar coating was inhibited. Serum dilutions were prepared in normal saline supplemented with antibiotics (streptomycin sulfate and benzylpenicillin G sodium salt), 100 U/ml each. The working dilution of the virus-containing suspension based on the SARS-CoV-2 virus was prepared in a Hanks’ balanced salt solution supplemented with 2% fetal bovine serum (FBS) and antibiotics. Concentration of SARS-CoV-2 in the prepared dilution was 100–150 PFU/ml (40–60 plaques per flask). A one-day-old monolayer of Vero C1008 cells in T25 flasks was used in the experiment. A mixture of equal volumes of the serum and SARS-CoV-2 virus culture was incubated at 37°C for 1 h. At least four flasks were used for each serum dilution. A mixture of the serum and virus culture (0.5 ml of each component) was placed in each flask, and the inoculum was uniformly distributed over the entire monolayer. The flasks were placed horizontally and left at 37°C. After adsorption of the antibodies–virus complex on the cells for 1 h, the inoculum was decanted, the primary agar coating designed for the SARS-CoV-2 virus was applied (10.0 ml per flask), and the monolayer was incubated at 37°C for two days. After the two days, a secondary agar coating containing a 0.1% Neutral Red solution was applied onto the infected monolayer for staining the cells and 24-hr incubation was performed at room temperature in the dark. Next, the negative colonies in the flasks were counted. The most dilute serum sample in which the formation of negative colonies by the SARS-CoV-2 virus was inhibited by at least 50% compared to the negative control (FBS containing no antibodies specific to the SARS-CoV-2 virus) was assumed to be the antibody titer in the analyzed serum sample.

**Determining the neutralizing activity in the pseudovirus system**

Testing with pVNT was performed using recombinant lentiviruses carrying the SARS-CoV-2 S protein and encoding firefly luciferase (Luc) [17]. To obtain pseudovirus particles, HEK293T cells were cultured in T75 flasks to a 50–70% confluence level and transfected with a mixture of plasmids (15 μg of pLuc, 15 μg of pGAG, 5 μg of pRev, and 2 μg of SARS-CoV-2 S protein per flask) using PEI (75 µg per flask) as a transfection agent. The cells were then incubated at 37°C, 5% CO_2_ for 72 h in the DMEM supplemented with 10% FBS. After the 72 h, the cell culture supernatant was centrifuged first at 150 g and then at 3,900 g, followed by filtration through a filter with a pore size of 0.20 μm. The resulting aliquots of the supernatant were stored at –80°C. The HEK293T-ACE2 cells were inoculated into 96-well plates at a density of 2 × 10^4^ cells/well and incubated overnight. Serial dilutions of serum samples in the DMEM medium supplemented with 10% FBS were prepared. The diluted serum samples (5 μl) were then mixed with the pseudovirus-containing medium (50 μl) in 96-well plates and incubated at 37°C, 5% CO_2_ for 1 h. Next, 50 μl of the medium was removed from the wells of the plates containing HEK293T-ACE2 cells and the cells were infected with virus–serum mixtures (50 μl/well). The inoculated HEK293T-ACE2 cells were then incubated at 37°C, 5% CO_2_ for 48 h. The controls were tested in three replicates; the analyzed samples were tested once. After the 48-h incubation, the medium was collected from the wells containing the cells; 100 μl of a lysing buffer (25 mM Tris-phosphate, pH 7.8, 1% Triton X-100, 10% glycerol, 2 mM DTT) was added into each well, and the plate was incubated for 5 min at room temperature. Next, 20 μl of the Bright-Glo™ Luciferase Assay Substrate reagent (Promega, USA) was added to the white 96-well plates containing 80 μl of the cell lysate, and the luminescence intensities were measured. The curves showing the luminescence intensity as a function of serum dilution were plotted using the GraphPad Prism 8 software, and serum titers ensuring 50% pseudovirus neutralization were calculated.

**Quantitative determination of RBD-specific neutralizing antibodies by competitive ELISA**

A PBS solution of recombinant RBD produced by expression of RBD (amino acid residues 320–537) in CHO cells was added into the wells of MaxiSorp 96-well plate (Nunc, Denmark) (100 μl) at a concentration of 1 μg/ml, and the plate was incubated overnight at 2–8°C. Next, the unoccupied binding sites were blocked by adding 150 μl of a blocking solution (PBS, 0.05% Tween-20, 0.1% BSA) into each well and incubating the plate at room temperature for 1 h. Serum samples in the blocking buffer were prepared in three dilutions (1 : 10, 1 : 50, and 1 : 250) and three replicates in a separate 96-well plate with a low sorption capacity. Solutions of the primary WHO standard and the secondary standard (obtained in the laboratory from the pool of serum samples collected from individuals who had had COVID-19 and characterized with respect to the primary standard) in the blocking buffer at final concentrations of 10, 20, and 40 IU/ml were prepared in the same plate. The analyzed serum samples and standards were then added into the wells of the plate containing the adsorbed RBD (100 μl/well) and incubated for 30 min in a thermo-shaker at 37°C, 700 rpm. After the incubation, the plate was washed five times by placing 350 μl of PBST (PBS, 0.05% Tween-20) into each well; 100 μl of the solution of recombinant hACE2-3×FLAG (0.2 μg/ml) in the blocking buffer was added into the wells. After 30-min incubation at 37°C, 700 rpm and washing, 100 μl of anti-FLAG antibodies conjugated to horseradish peroxidase (Sigma Aldrich, USA, Cat. # A8592) at a 1 : 10 000 dilution in the blocking buffer were added into each well and the plate was incubated for an additional 30 min using the procedure described above. After the plate had been washed, 100 μl of the TMB substrate solution was added into each well and the plate was incubated in the dark for 15 min. The enzymatic reaction was stopped by adding a 10% orthophosphoric acid solution, and the OD_450_ values in the wells were measured on a spectrophotometer plate reader. The curves showing OD_450_ as a function of the concentration of RBD-specific neutralizing antibodies in IU standards (IU/ml) were plotted using the GraphPad Prism 8 software. These curves were used to calculate the concentrations of RBD-specific neutralizing antibodies in the serum samples; for this purpose, a dilution of the sample that laid in the range of OD_450_ values of the standard curve was selected and the obtained value in IU/ml was multiplied by the respective dilution. The detection limit was determined as follows: the mean OD_450_ value in the negative samples minus three standard deviations from the mean in the negative samples.

## RESULTS

**Developing the ELISA kit for a quantitative determination of the SARS-CoV-2 S1 RBD-neutralizing activity in human sera**

There are several methods for a quantitative determination of the virus-neutralizing activity of serum samples: the standard assay with live viruses (cVNT), the assay with pseudoviruses (pVNT), and the competitive ELISA assay, which is based on immunochemical methods (sVNT). The standard “live” virus assays (in the case of SARS-CoV-2) need to be performed indoors, in facilities with a biosafety level no lower than BSL-3. Assays involving pseudoviruses (PV) are labor-intensive and time-consuming. Competitive ELISA assays are convenient for routine serodiagnosis and take comparatively less time. However, they need to be validated with respect to other types of assays.

**Fig. 1:**
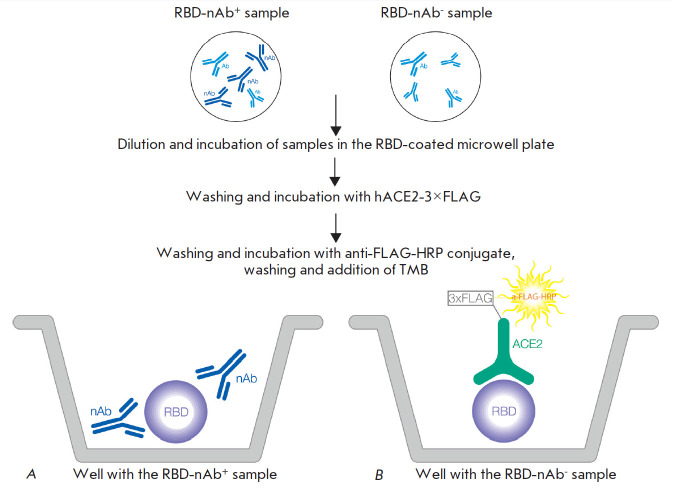
Scheme of the quantitative determination of the activity of SARS-CoV-2 RBD-specific neutralizing antibodies in serum or plasma (sVNT). Antibodies in the serum sample interact with the recombinant RBD adsorbed in the wells. If the sample contains RBD-neutralizing antibodies (*A*), they block the binding of RBD to ACE2. If the sample does not contain neutralizing antibodies (*B*), the RBD adsorbed on the plate binds to recombinant ACE2. This binding is detected by peroxidase-labeled antibodies against the 3×FLAG sequence (3×FLAG) contained in recombinant ACE2. Therefore, the colorimetric signal recorded in the assay is inversely proportional to the concentration of the neutralizing antibodies in the sample. Designations: Ab – antibodies without neutralizing activity; ACE2 – recombinant human ACE2 receptor; HRP – antibodies to the FLAG epitope labeled with horseradish peroxidase; nAB – antibodies with RBD-neutralizing activity; RBD – the recombinant receptor-binding domain of the coronavirus SARS-CoV-2 S protein

We have developed an ELISA kit for a quantitative determination of the activity of SARS-CoV-2 RBD-specific neutralizing antibodies in serum or plasma [18]. The method is based on a competitive enzyme-linked immunosorbent assay (sVNT) for measuring the interaction between the recombinant receptor-binding domain (RBD) of the surface glycoprotein (S protein) of the SARS-CoV-2 coronavirus and the recombinant human ACE2 receptor (ACE2), in the presence of the analyzed sample. During the first stage, SARS-CoV-2 RBD-neutralizing antibodies (if present in the analyzed samples) interact with RBD adsorbed on the surface of the wells of a dismountable polystyrene plate. During the second stage, the RBD interacts with the human recombinant ACE2 receptor. If the analyzed sample contains no RBD-neutralizing antibodies, the RBD–ACE2 complex appears. If the sample contains RBD-neutralizing antibodies, the RBD–ACE2 complex is formed either partially or not at all. The resulting RBD–ACE2 complex is detected using an immunoenzyme conjugate at the third stage (Fig. 1).

The total time needed to perform the assay is 2–2.5 h. The international WHO standard is used for detection; the detection limit is 4 IU/ml.

**The RBD-neutralizing activity of serum samples measured by competitive ELISA assay strongly correlates with virus neutralization**

**Fig. 2:**
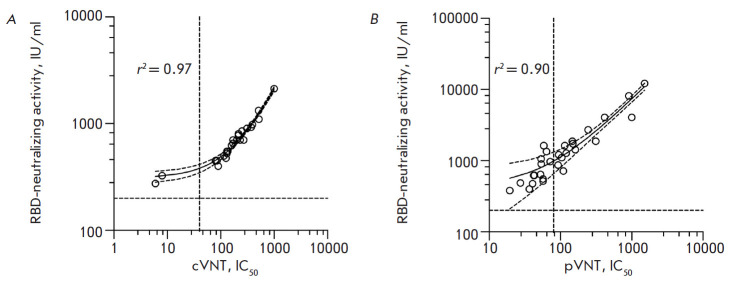
Validation of the RBD neutralization test by comparison with the conventional and pseudovirus neutralization assays. (*A*) Plot showing the activity of SARS-CoV-2 RBD-specific neutralizing antibodies in sera obtained by sVNT against serum titers that yield 50% virus neutralization (IC_50_) using the cVNT test (26 samples). (*B*) Plot showing the activity of SARS-CoV-2 RBD-specific neutralizing antibodies in sera obtained by sVNT against sera titers at which pseudovirus neutralization of 50% (IC_50_) was achieved (pVNT test) (29 samples). r2 is the coefficient of determination. In all the serum samples where no anti-RBD neutralizing antibodies were detected (25 samples), neutralization of the SARS-CoV-2 infection was not detected in all the tests

We have performed a successful validation of the designed competitive ELISA kit by comparing the RBD-neutralizing activity data to the virus neutralization data obtained using both standard testing with “live” viruses (cVNT) and testing with pseudoviruses (pVNT). The fidelity parameters of linear approximation (r2) were 0.97 and 0.90, respectively (Fig. 2).

**Characterizing the groups of serum samples and analyzing their protective properties**

We analyzed 134 serum samples obtained from four groups of individuals (Table): patients who had suffered severe COVID-19 (Hospitalized patients); patients who had had mild COVID-19 (Outpatients); individuals who had not previously had COVID-19 and had been vaccinated with two doses of Gam- COVID-Vac (Vaccinated with Gam-COVID-Vac); and individuals who had not previously had COVID-19 and had been vaccinated with two doses of CoviVac (Vaccinated with CoviVac).

All the serum samples were analyzed using both the developed sVNT method, which determines the SARS-CoV-2 RBD-neutralizing antibodies (RBD-nAb) activity – the ability of sera to inhibit (neutralize) RBD–ACE2 binding – and our in-house quantitative ELISA kit, which determines the total concentration of SARS-CoV-2 RBD-specific immunoglobulins G (IgG). In the latter case, quantification is also performed with respect to the international WHO standard; the detection limit is 1 BAU/ml.

**Fig. 3:**
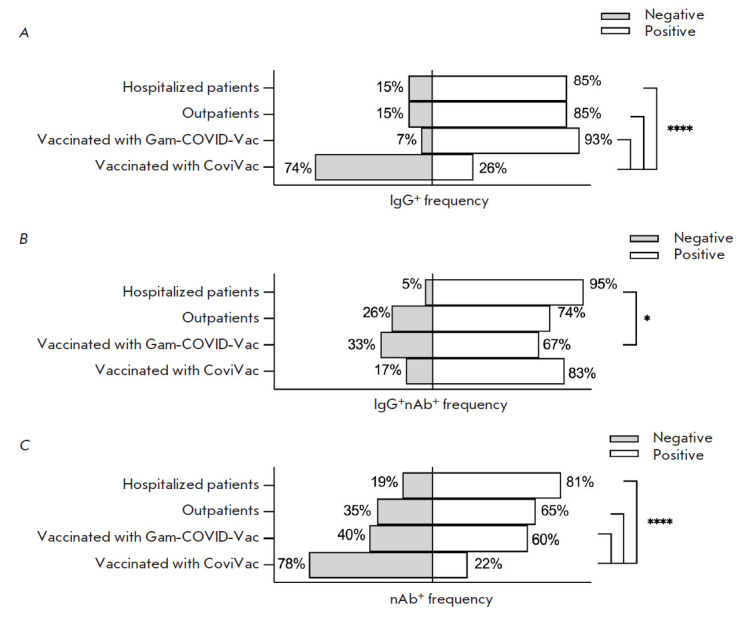
Frequency of seropositive serum samples in the analyzed groups. (*A*) Frequency of anti-RBD IgG positive serum samples per group. (*B*) Frequency of RBD-nAb seropositive serum samples among RBD-IgG-positive samples. (*C*) Frequencies of occurrence of RBD-nAb-positive samples in the groups. Statistical significance of the intergroup differences was determined using the Fisher’s exact test (* *p * < 0.05; **** *p * < 0.0001)

**Table:** The analyzed serum sample groups

Group	Number	Sex, males/females	Age, median (minimum, maximum)	Time (days) after the symptom onset or injection of the second vaccine dose, median (minimum, maximum)
Hospitalized patients	27	15/12	57 (37, 69)	23 (19, 47)
Outpatients	41	21/20	39 (27, 61)	25 (17, 44)
Vaccinated with Gam-COVID-Vac	43	20/23	41 (25, 62)	21 (14, 28)
Vaccinated with CoviVac	23	11/12	36 (28, 58)	20 (14, 30)

The frequency of occurrence of IgG-positive sera samples among the groups Hospitalized patients, Outpatients, and Vaccinated with Gam-COVID-Vac varied from 85 to 93%. In the group Vaccinated with CoviVac, the frequency of occurrence was as low as 26% (Fig. 3A). Among these IgG-seropositive sera samples, the frequency of occurrence of RBD-nAb-positive serum samples varied from 67 to 95% (Fig. 3B). The frequency of occurrence of RBD-nAb-positive serum samples in the group (showing the protective properties of the serum samples in the group) varied from 22 to 81% (Fig. 3C).

**Fig. 4:**
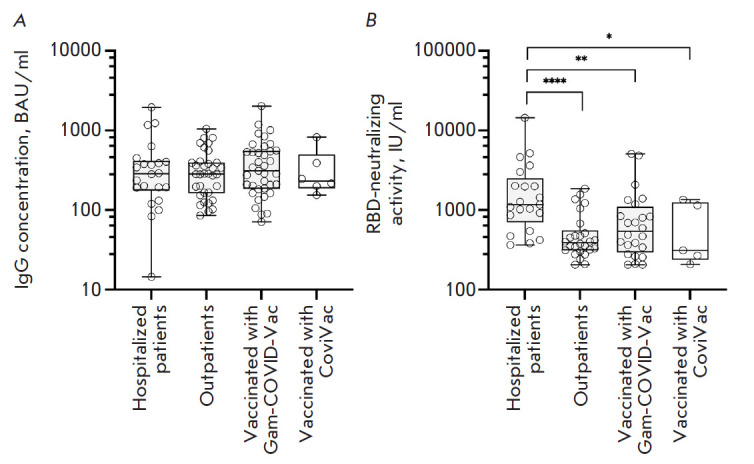
Concentration of SARS-CoV-2 RBD-specific IgG and the activity of RBD-ACE2 neutralizing antibodies in seropositive serum samples measured by sVNT. (*A*) Concentration of SARS-CoV-2 RBD-specific IgG in seropositive serum samples. (*B*) Neutralizing activity of RBD-specific antibodies (RBD-nAb) in seropositive serum samples. The statistical significance of intergroup differences was determined using the Kruskal–Wallis test (* *p * < 0.05; ** *p* < 0.01; **** *p * < 0.0001)

The concentrations of SARS-CoV-2 RBD-specific IgG in the seropositive samples in different groups varied insignificantly (Fig. 4A). However, although the concentrations of RBD-specific IgG in the seropositive samples were almost identical for all the groups, the activity of RBD-specific virus-neutralizing antibodies was substantially higher in the group Hospitalized patients compared to those in the other groups (Fig. 4B).

**Fig. 5:**
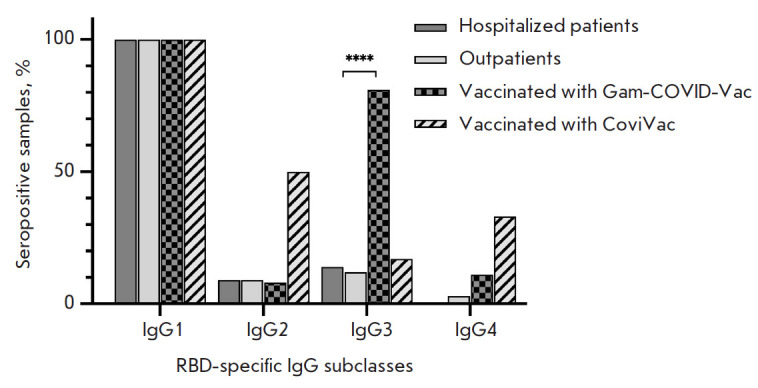
The frequency of occurrence of SARS-CoV-2 RBD-specific immunoglobulin class G subclasses among RBD-nAb+ samples. Statistical significance of intergroup differences was determined using the Fisher’s exact test (* *p* < 0.05; **** *p * < 0.0001)

In order to further elucidate the nature of the humoral response, the double-positive (RBD-IgG+ and RBD-nAb+) samples were tested using subtype-specific conjugates. An analysis of IgG subclasses revealed an increased production of IgG3 antibodies in the group of individuals vaccinated with Gam-COVID-Vac, along with a switch to the IgG1 subclass in all the groups (Fig. 5).

**The relationship between the activity of RBD-neutralizing antibodies and concentration of anti-RBD IgG**

**Fig. 6:**
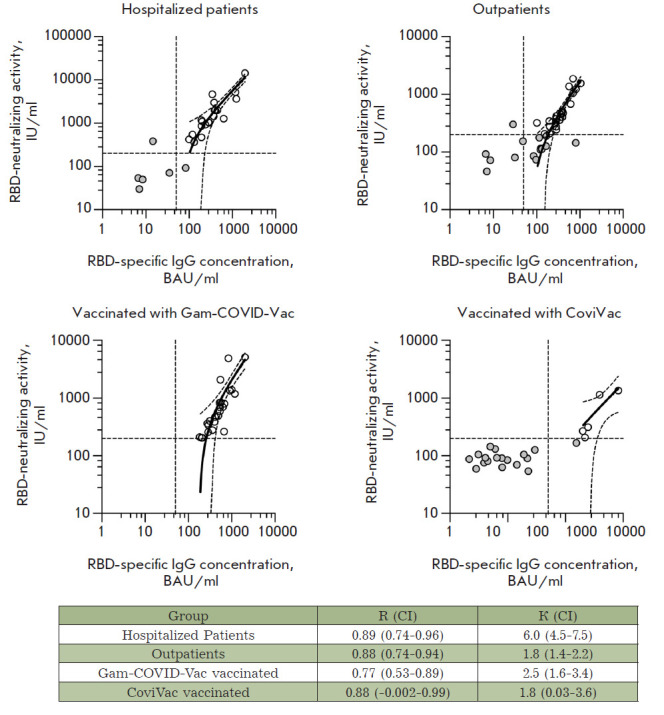
Linear regression analysis of the serum antibody RBD-ACE2 neutralizing activity and RBD-specific IgG concentration. Double seropositive (RBD-IgG+ and RBD-nAb+) serum samples are shown as white circles; negative samples are shown as gray circles. The 95% confidence intervals and activity and concentration thresholds are shown with dotted lines. **R **is the Pearson correlation coefficient; **K **is the slope of the regression line; **CI **is the 95% confidence interval

In order to characterize the relationship between the activity of SARS-CoV-2 RBD-specific nAb and the concentration of SARS-CoV-2 RBD-specific IgG, we conducted a linear regression analysis of each group of serum samples. Differences in RBD-nAb activity (normalized with respect to the concentration of RBD - specific IgG) were revealed in the analyzed samples from different groups. The activity of RBD-nAb can be expressed as a slope of the regression line (Fig. 6). The activity of RBD-nAb was significantly higher in the serum samples of the group Hospitalized patients compared to the other groups.

**The RBD-neutralizing properties of serum samples collected from individuals in different groups**

**Fig. 7:**
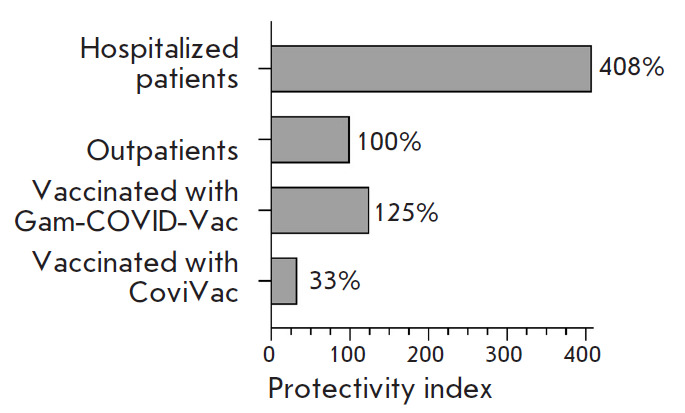
The virus-neutralizing activity of the humoral immunity in the study groups. The protectivity index of the group *Outpatients *was taken as 100%

To perform an adequate assessment of the RBD-neutralizing activity of the humoral immunity (the protectivity index), one needs to take into account, along with the activity of RBD-neutralizing antibodies, the frequency of immune response formation in the analyzed group. Therefore, the protectivity index of sera in the different groups was calculated as the slope of the regression line (K) in the group (Fig. 6) normalized to the frequency of occurrence of SARS-CoV-2 RBD-specific nAb-positive serum samples in the group (Fig. 3C). The resulting data are shown in Fig. 7.

## DISCUSSION

Emergence, development, and persistence of humoral immunity to the SARS-CoV-2 coronavirus in patients who have recovered after COVID-19 and/or had been vaccinated are extremely important and largely inform the measures taken by the state in combatting the coronavirus infection. Neutralizing antibodies play a significant role in protecting the or ganism against the virus. The mechanisms of action of virus-neutralizing antibodies are rather diverse and involve the inhibition of virion binding to cellular receptors, inhibition of penetration of the viral genomes into the cytoplasm, blocking of the penetration of the viral genomes from the endosome into the cytoplasm, and, finally, simple aggregation of viral particles. The main type of neutralizing antibodies in patients with the SARS-CoV-2 infection are those preventing the interaction between the receptor-binding domain of the virus S protein to the ACE2 cell receptor. A large number of studies showing a correlation between the protection level and the presence of SARS-CoV-2 anti-RBD immunoglobulins G in human serum have been conducted [19 , 20, 21, 22].

A particular pool of studies has focused on adaptive immunotherapy of COVID-19; namely, on designing recombinant therapeutic virus-neutralizing antibodies against SARS-CoV-2 [23, 24, 25]. In this case, the potential protection against new virus variants is of particular interest and there is also much tension around the issue of assaying virus neutralization. For SARS-CoV-2, to some extent, it is fair to say that the RBD-ACE2-neutralizing activity of sera is almost equivalent to the virus-neutralizing activity of antibodies and can be used as an analog to assay neutralizing antibodies against SARS-CoV-2.

The objective of this study was to thoroughly investigate the protective properties of the humoral immune response in hospitalized patients and outpatients, as well as individuals who have received prophylaxis with the two vaccines against SARS-CoV-2 which are the most popular in the Russian Federation.

To study the humoral immunity against SARS-CoV-2, we have designed two simple, quick and convenient-to-use ELISA kits: for a quantitative determination of the SARS-CoV-2 anti-RBD-IgG concentration and for a quantitative determination of the SARS-CoV-2 S1 RBD-ACE2-neutralizing activity of antibodies (RBD-nAb). These kits form a platform, that, owing to their modular structure, within a short period of time (up to 1–2 weeks) can be adapted to new strains (by replacing the RBD of the protein) or even to new viruses (by replacing the ACE2 receptor).

We used these kits to determine the following parameters of the blood serum samples for the analyzed groups of patients: the frequency of occurrence of SARS-CoV-2 anti-RBD-IgG positive sera, IgG concentration in serum samples, the frequency of occurrence of SARS-CoV-2 RBD-nAb-positive sera, and the level of neutralizing activity of RBD-nAb in serum samples.

The concentrations of SARS-CoV-2 anti-RBD-IgG antibodies were almost identical for all the seropositive serum samples; however, the frequency of occurrence of IgG-positive sera in the group of individuals vaccinated with CoviVac based on the inactivated virus was more than threefold lower compared to the remaining groups. Earlier, we have demonstrated that most SARS-CoV-2 anti-RBD antibodies in patients who had had COVID-19 were conformationally dependent [16, 26]. CoviVac apparently has an appreciably low immunogenicity, which is probably caused by partial disruption of the structure of the S-protein epitopes during virus inactivation or storage. The frequency of occurrence of RBD-nAb-positive sera, as well as their activity, was highest in the group Hospitalized patients.

We also studied the profile of formation of IgG-antibody subclasses in different groups. IgG1 antibodies were detected in serum samples in all the groups. Notably, the group Vaccinated with Gam-COVID-Vac contained also antibodies of the IgG3 subclass. Switching to the production of IgG1 and IgG3 subclasses antibodies seems to be induced by IL-21 [27]. IgG3 antibodies are formed at the early stages of the immune response and are characterized by a high ability to activate complement and high affinity to Fcγ cellular receptors. In addition to the RBD-neutralizing activity, all the aforelisted properties of antibodies of this subclass trigger the activation of antibody-dependent cellular phagocytosis and antibody-dependent cytotoxicity [28 , 29, 30]. The switching to the production of antibodies of the IgG1 and IgG3 subclasses in the group Vaccinated with Gam-COVID-Vac can be explained by the nature of the adenoviral vector used in the Gam-COVID-Vac vaccine.

We have also calculated the RBD-neutralizing activity of the humoral immunity in the analyzed groups. The protectivity index in the group Vaccinated with Gam-COVID-Vac was higher than that in the group Outpatients by 25% and higher than that in the group Vaccinated with CoviVac almost fourfold. The highest RBD-neutralizing activity was observed in the group Hospitalized patients (fourfold higher compared to the group Outpatients), being indicative of the presence of high-affinity and high-specificity antibodies, along with a high frequency of development of humoral immunity. This fact can be attributed to the long-term viral load in hospitalized patients, which leads to the development of virus-neutralizing antibodies with a high affinity to the viral epitopes [16, 31, 31, 32, 33].

## CONCLUSIONS

A relevant platform for the quantitative analysis of RBD-neutralizing antibodies against SARS-CoV-2 has been designed as an alternative to monitoring the virus-neutralizing activity, making it possible to quantify the concentrations of SARS-CoV-2 anti-RBD IgG antibodies, as well as the SARS-CoV-2 RBD-ACE2- neutralizing activity of the antibodies.

A comparative study of 134 serum samples collected from patients who had suffered severe and mild COVID-19 and individuals vaccinated with Gam- COVID-Vac and CoviVac was performed.

The highest protectivity index was observed in the group Hospitalized patients.

The protective properties of humoral immunity after vaccination with Gam-COVID-Vac was fourfold stronger than that after vaccination with CoviVac.

The advantage of the developed platform is that it allows one to adapt the method to newly emerging virus variants in the shortest possible period of time (1–2 weeks) and, thereby, collect quantitative data on the protection level afforded individuals vaccinated with earlier types of vaccines.
